# Trends in Nonsurgical Management for Low-Risk, Hormone Receptor–Positive Ductal Carcinoma In Situ

**DOI:** 10.1001/jamanetworkopen.2025.58248

**Published:** 2026-02-10

**Authors:** Yu Matsui, Jincong Q. Freeman, Sarah Poland, Frederick M. Howard, Nan Chen, Olufunmilayo I. Olopade, Dezheng Huo

**Affiliations:** 1 Center for Innovation in Global Health, The University of Chicago, Chicago, Illinois; 2 Department of Dermatology, Faculty of Medicine, Academic Assembly, University of Toyama, Toyama, Japan; 3 Department of Public Health Sciences, The University of Chicago, Chicago, Illinois; 4 Cancer Prevention and Control Research Program, University of Chicago Medicine Comprehensive Cancer Center, Chicago, Illinois; 5 Center for Health and the Social Sciences, The University of Chicago, Chicago, Illinois; 6 Section of Hematology/Oncology, Department of Medicine, The University of Chicago, Chicago, Illinois

## Abstract

**Importance:**

Active surveillance has emerged as a deescalation strategy for low-risk ductal carcinoma in situ (DCIS) to reduce overtreatment while maintaining favorable outcomes. Emerging data in low-risk DCIS, eg, the COMET trial, have highlighted growing interest in surveillance-based management for carefully selected patients. However, recent clinical adoption and national trends in managing low-risk, hormone receptor (HR)–positive DCIS have not been evaluated in the US.

**Objective:**

To examine trends and sociodemographic variations in nonsurgical management and other treatment modalities for low-risk, HR-positive DCIS.

**Design, Setting, and Participants:**

This cross-sectional study analyzed data from the National Cancer Database from January 1, 2004, to December 31, 2022, and included patients aged 18 years or older with grade 1 to 2, HR-positive DCIS and at least 12 months of follow-up since initial diagnosis. Analyses were performed between January 10 and August 31, 2025.

**Exposures:**

Year of diagnosis and sociodemographic characteristics.

**Main Outcomes and Measures:**

Nonsurgical management, lumpectomy alone, lumpectomy plus adjuvant radiotherapy, unilateral mastectomy, bilateral mastectomy, and endocrine therapy were measured using descriptive statistics.

**Results:**

A total of 316 590 female patients were included (mean [SD] age, 60.8 [12.0] years; 5.8% Asian or Pacific Islander, 13.9% Black, 6.1% Hispanic, 73.3% White, and 0.9% other race and ethnicity). From 2004 to 2022, nonsurgical management increased from 2.1% to 3.5%, bilateral mastectomy increased from 4.1% to 8.7%, and lumpectomy increased from 22.0% to 25.1%, while lumpectomy plus adjuvant radiotherapy decreased from 50.9% to 45.6% and unilateral mastectomy decreased from 20.9% to 17.1%. Nonsurgical management was more common among Black patients and patients with no insurance. Bilateral mastectomy was common in younger, White, and privately insured patients and those who lived in higher-income areas. Endocrine therapy use increased from 2004 to 2020 but declined thereafter. Endocrine therapy was highest after lumpectomy plus adjuvant radiotherapy (69.6%), followed by lumpectomy alone (43.9%), unilateral mastectomy (35.3%), and nonsurgical management (29.2%), with the lowest use in patients younger than 50 years in the no surgery (15.2%) and lumpectomy alone (38.6%) groups. Since 2018, radiotherapy use has increased and become progressively more risk adapted, with increasing use with higher Oncotype DX DCIS scores (low risk, 34.5%; intermediate risk, 63.9%; high risk, 73.1%).

**Conclusions and Relevance:**

This cross-sectional study highlights increasing trends and socioeconomic disparities in the nonsurgical management of and the need for precision-based, patient-centered care for low-risk DCIS. Precision prevention may enhance the identification of patients who could benefit most from preventive surgery, prolonged endocrine therapy, or treatment deescalation, paving the way for individualized strategies.

## Key Points

**Question:**

What are the trends and patterns of nonsurgical management and other treatment modalities for low-risk, hormone receptor–positive ductal carcinoma in situ (DCIS) in the US?

**Findings:**

In this cross-sectional study of 316 590 patients with grade 1 to 2 DCIS, nonsurgical management and bilateral mastectomy increased from 2004 to 2022, adjuvant radiotherapy became genomic risk adapted since 2018, and endocrine therapy increased from 2004 to 2020 but declined thereafter. Sociodemographic variations were observed across treatment modalities.

**Meaning:**

These findings highlight growing heterogeneity in DCIS management and the need for precision-prevention frameworks that align treatment intensity with cancer risk, patient preferences, and evolving evidence.

## Teaser

This cross-sectional study examines trends and sociodemographic variations in nonsurgical management and other treatment modalities for low-risk, hormone receptor–positive ductal carcinoma in situ in the US.

## Introduction

Ductal carcinoma in situ (DCIS) represents a noninvasive precursor to breast cancer, characterized by the proliferation of neoplastic epithelial cells confined to the mammary ductal system without invasion of the basement membrane.^1^ The widespread adoption of screening mammography has led to a marked increase in DCIS detection,^2,3^ estimated to have affected 56 500 women annually in the US in 2024.^4^ Studies have shown that approximately 20% to 40% of untreated cases of DCIS progress to invasive breast cancer over a 10-year period, raising concerns regarding optimal management and treatment strategies.^5,6^ The majority of patients with DCIS are treated with surgical resection, often followed by adjuvant radiotherapy and/or endocrine therapy, generally using less-intensive regimens than those used for invasive disease.^7^ However, the indolent nature of many low-grade, hormone receptor (HR)–positive DCIS lesions, particularly those detected through screening, has led to growing interest in deescalation strategies, including protocol-driven active surveillance used in clinical trials and broader nonoperative care in clinical settings.^8^ In this study, we operationalized this broader nonoperative care in the National Cancer Database (NCDB) as the no surgery or nonsurgical management category. Risk-stratifying genomic assays also have been developed in recent years, with the Oncotype DX (ODX) DCIS score being the most widely used. Designed to refine postlumpectomy recurrence risk and guide the omission of adjuvant radiotherapy, its clinical adoption and impact remain scarce and inconclusive at the national level.^9,10^

Four ongoing randomized clinical trials, COMET in the US,^11^ LORIS in the UK,^12^ LORD in the Netherlands,^13^ and LORETTA in Japan,^14^ were launched to evaluate the safety and acceptability of protocol-defined active surveillance in patients with low-risk DCIS. The COMET trial has demonstrated that active surveillance, with breast imaging and physical examination every 6 months, does not result in a higher rate of ipsilateral invasive breast cancer at 2 years compared with guideline-concordant care consisting of surgery with or without radiotherapy.^15^ These results suggest that active surveillance may offer a safe alternative to surgery and radiotherapy for select patients, potentially reducing overtreatment without compromising outcomes.^15^

Using the 2004-2022 NCDB, we quantified nationwide trends in nonsurgical management and other treatment modalities for low-risk, HR-positive DCIS to clarify how management of low-risk disease has evolved amid growing interest in deescalation.^16^ In this study, nonoperative cases were defined using established NCDB-based criteria commonly applied in prior studies and encompassing treatment refusal, medical contraindications, delayed surgery, and other nonoperative scenarios rather than trial protocol–driven active surveillance. Understanding actual treatment approaches is essential to evaluate whether patients are receiving care consistent with the clinical characteristics and prognostic risk of their low-risk DCIS. The goal of this study was to provide insight into the evolving landscape of DCIS management and to inform future efforts toward more personalized and risk-adapted care.

## Methods

### Design, Setting, and Participants

This cross-sectional study analyzed data collected from patients diagnosed with DCIS or invasive breast cancer between January 1, 2004, and December 31, 2022, from the NCDB. The NCDB, a joint project of the Commission on Cancer of the American College of Surgeons and the American Cancer Society,^17^ is a large clinical oncology registry that captures approximately 72% of new cancer diagnoses from more than 1500 Commission on Cancer–accredited programs in the US each year.^18,19,20^ As the NCDB contains only deidentified data, The University of Chicago Institutional Review Board deemed this study exempt from review and waived the requirement for informed consent. The study adhered to the Strengthening the Reporting of Observational Studies in Epidemiology (STROBE) reporting guideline.^21^

**Figure 1.**
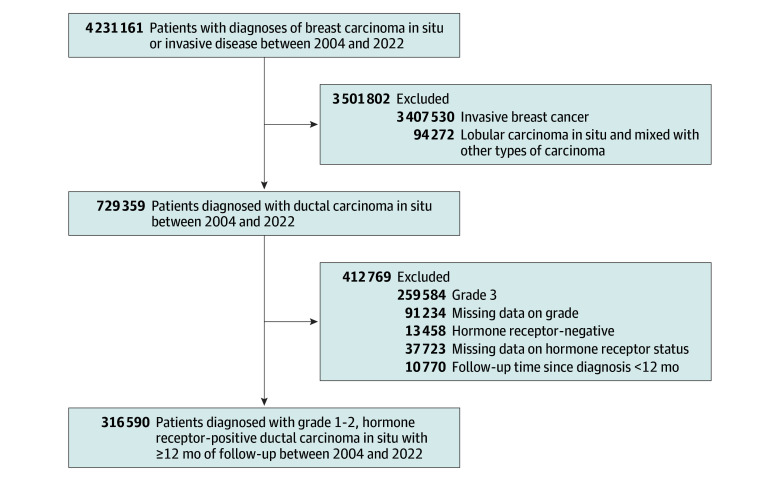
Consolidated Standards of Reporting Flow Diagram for Cohort Selection of Low-Risk, Hormone Receptor–Positive Ductal Carcinoma In Situ From the 2004-2022 National Cancer Database

We used the NCDB 2022 Participant User File to identify patients aged 18 years or older who were diagnosed with DCIS between 2004 and 2022. Patients with invasive breast cancer, missing diagnoses, or lobular carcinoma in situ were excluded. Patients with grade 3 disease, unknown tumor grade or HR status, HR-negative tumors, and less than 12 months of follow-up were further excluded, yielding a final sample of patients with grade 1 to 2, HR-positive DCIS (Figure 1).

### Outcomes and Measures

The outcomes of interest were nonsurgical management and other treatment modalities for DCIS. Patients were categorized into 5 mutually exclusive initial local therapy categories based on the following NCDB surgery and radiotherapy variables: no surgery, lumpectomy alone, lumpectomy plus adjuvant radiotherapy, unilateral mastectomy, and bilateral mastectomy. In this study, the no surgery group was defined using established NCDB-based criteria commonly applied in previous observational studies evaluating nonoperative management. Specifically, patients were classified into this group if (1) surgery was not performed because it was not part of a planned initial course of treatment; it was not recommended due to contraindication toward patients’ risks (eg, comorbidities, older age, disease progression prior to surgery); or it was declined by the patients, their family members, or other guardians or (2) surgery was performed more than 365 days after the initial diagnosis. These criteria operationalized nonoperative management within the NCDB but did not represent protocol-driven active surveillance as evaluated in clinical trials. For clarity and to avoid misinterpretation, we refer to this NCDB-defined group as no surgery or nonsurgical management throughout this report. The numerical ODX DCIS score became available in the NCDB in 2018 and was classified into 3 risk categories: low risk (<39), intermediate risk (39-54), and high risk (≥55). Endocrine therapy use was ascertained and summarized both overall and within each local therapy category. Patients who underwent bilateral mastectomy were excluded from the endocrine therapy analyses because removal of all breast tissue leaves minimal residual risk, and endocrine therapy is not the standard of care in these patients.^22^

### Key Variables and Covariates

Key variables collected included year of initial DCIS diagnosis (2004-2022) and race and ethnicity. We categorized race and ethnicity as Hispanic, non-Hispanic Asian or Pacific Islander (hereafter, Asian or Pacific Islander), non-Hispanic Black (hereafter, Black), non-Hispanic White (hereafter, White), and other. Other is a predefined NCDB category that includes individuals classified by facility registrars as American Indian, Alaska Native, other race, or unknown race and ethnicity. Race and ethnicity were included to assess potential differences in treatment patterns and outcomes that may reflect disparities in access to care, health care delivery, and structural factors rather than biological differences. Additional sociodemographic variables were age at diagnosis, type of insurance (Medicaid, Medicare, private, other, or uninsured), area-level education, median household income quartiles, and residential area (metropolitan, rural, or urban). As a proxy for community-level educational attainment, area-level education was defined as the percentage of adults aged 25 years or older in the patient’s residential zip code who did not graduate from high school according to 2020 American Community Survey data. Median household income quartiles were classified based on 2020 American Community Survey data, spanning the years 2016 to 2020 while adjusting for 2016 inflation. Clinicopathologic covariates included tumor grade, progesterone receptor status, and the Charlson-Deyo Comorbidity Index (0, 1, and ≥2). Facility type was recorded based on Commission on Cancer–accredited program designation, classified as community, comprehensive community, academic or research, and integrated network.

### Statistical Analysis

Descriptive statistics were used to summarize patient characteristics, both overall and by treatment group. Categorical variables were compared using χ^2^ tests, and continuous variables were compared using analysis of variance or Kruskal-Wallis test, as appropriate. Annual proportions of patients undergoing nonsurgical treatment and other treatment modalities were calculated from 2004 to 2022. Endocrine therapy use was summarized across treatment groups, excluding bilateral mastectomy. Radiotherapy use was analyzed by ODX DCIS risk group between 2018 and 2022, when the NCDB began collecting this genomic information. Temporal trends in treatment use were assessed using the generalized linear model, with a log-link and binomial distribution. Percentage change per year and 95% CIs were estimated. All statistical tests were 2-sided, with a *P* < .05 considered statistically significant. All analyses were performed between January 10 and August 31, 2025, using Stata, version 17.0 (StataCorp LLC).

## Results

### Patient Characteristics

Of 4 231 161 patients with breast cancer in the NCDB, 3 914 571 were excluded, leaving a total of 316 590 female patients with grade 1 to 2, HR-positive DCIS (mean [SD] age, 60.8 [12.0] years; 5.8% identified as Asian or Pacific Islander, 13.9% as Black, 6.1% as Hispanic, 73.3% as White, and 0.9% as other race and ethnicity). A total of 45.5% of patients resided in areas with a median household income of $74 063 or higher; 55.3% had private insurance and 37.2% Medicare; 72.8% were diagnosed with tumor grade 2; 92.6% had progesterone receptor–positive disease; and 83.9% had no comorbidities (Table).

**Table.** Sociodemographic and Clinicopathologic Characteristics of Patients With Low-Risk, Hormone Receptor–Positive Ductal Carcinoma In Situ

Characteristic	Patients, No. (%)						*P* value^a^
	Overall	No surgery	Lumpectomy	Lumpectomy plus radiotherapy	Unilateral mastectomy	Bilateral mastectomy	
Total	316 590 (100)	7876 (2.6)	73 604 (24.2)	139 705 (46.0)	56 446 (18.6)	26 101 (8.6)	NA
Age at diagnosis, y							
Mean (SD)	60.8 (12.0)	64.7 (14.0)	65.4 (12.1)	60.2 (10.7)	59.2 (12.4)	53.2 (10.9)	<.001
Median (IQR)	61.0 (51.0-70.0)	65.0 (54.0-75.0)	67.0 (57.0-74.0)	61.0 (52.0-68.0)	59.0 (49.0-69.0)	52.0 (45.0-61.0)	<.001
Race and ethnicity							
Asian or Pacific Islander	18 212 (5.8)	473 (6.1)	3941 (5.4)	7802 (5.7)	4115 (7.4)	1179 (4.6)	<.001
Black	43 574 (13.9)	1577 (20.5)	9531 (13.1)	18 830 (13.6)	8911 (15.9)	2630 (10.2)	
Hispanic	19 128 (6.1)	508 (6.6)	4327 (5.9)	8327 (6.0)	3726 (6.7)	1324 (5.1)	
White	229 879 (73.3)	5034 (65.4)	54 504 (74.7)	102 387 (73.9)	38 641 (69.0)	20 495 (79.2)	
Other^b^	2889 (0.9)	102 (1.3)	642 (0.9)	1162 (0.8)	591 (1.1)	257 (1.0)	
No high school degree quartile^c^							
First (≥15.3%)	47 133 (17.2)	1511 (22.1)	10 858 (17.1)	20 185 (16.8)	9224 (19.0)	3107 (13.8)	<.001
Second (9.1%-15.2%)	69 517 (25.4)	1733 (25.3)	16 019 (25.2)	31 113 (25.9)	12 531 (25.7)	5459 (24.3)	
Third (5.0%-9.0%)	81 731 (29.9)	1900 (27.7)	19 111 (30.1)	36 674 (30.5)	14 162 (29.1)	6859 (30.5)	
Fourth (<5.0%)	74 927 (27.4)	1704 (24.9)	17 549 (27.6)	32 344 (26.9)	12 751 (26.2)	7041 (31.3)	
Median household income quartile^d^							
First (<$46 277)	35 879 (13.2)	1095 (16.0)	7880 (12.4)	15 750 (13.1)	7165 (14.8)	2468 (11.0)	<.001
Second ($46 227-$57 856)	49 564 (18.2)	1224 (17.9)	11 359 (17.9)	22 606 (18.8)	9002 (18.5)	3754 (16.7)	
Third ($57 857-$74 062)	62 429 (22.9)	1563 (22.9)	14 434 (22.8)	28 075 (23.4)	10 906 (22.5)	5105 (22.8)	
Fourth (≥$74 063)	124 912 (45.8)	2954 (43.2)	29 731 (46.9)	53 656 (44.7)	21 497 (44.3)	11 108 (49.5)	
Type of health insurance							
None	4178 (1.3)	183 (2.4)	763 (1.0)	1873 (1.4)	878 (1.6)	289 (1.1)	<.001
Private	172 818 (55.3)	3282 (42.7)	31 554 (43.4)	79 801 (57.7)	31 787 (57.2)	19 289 (74.6)	
Medicaid	15 798 (5.1)	459 (6.0)	2915 (4.0)	7297 (5.3)	3234 (5.8)	1220 (4.7)	
Medicare	116 461 (37.2)	3668 (47.7)	36 886 (50.7)	47 666 (34.5)	19 030 (34.2)	4684 (18.1)	
Other	3434 (1.1)	96 (1.2)	635 (0.9)	1548 (1.1)	660 (1.2)	371 (1.4)	
Residential area^e^							
Metropolitan	268 957 (87.6)	6874 (90.0)	62 948 (88.2)	118 400 (87.2)	47 593 (87.2)	21 855 (86.9)	<.001
Urban	32 835 (10.7)	679 (8.9)	7240 (10.1)	15 067 (11.1)	6075 (18.5)	2833 (11.3)	
Rural	5116 (1.7)	82 (1.1)	1150 (1.6)	2383 (1.8)	884 (1.6)	458 (1.8)	
Facility type or cancer program							
Community	19 353 (6.3)	512 (6.7)	4531 (6.2)	9622 (7.0)	2972 (5.5)	1018 (4.2)	<.001
Comprehensive community	125 434 (40.6)	2869 (37.3)	28 898 (39.7)	57 595 (41.8)	21 169 (39.0)	9969 (41.4)	
Academic or research	94 580 (30.6)	2799 (36.4)	22 013 (30.2)	39 769 (28.9)	17 786 (32.8)	7225 (30.0)	
Integrated network	69 853 (22.6)	1510 (19.6)	17 337 (23.8)	30 829 (22.4)	12 306 (22.7)	5895 (24.5)	
Charlson-Deyo Comorbidity Index							
0	265 662 (83.9)	6854 (87.0)	60 666 (82.4)	118 262 (84.7)	46 416 (82.2)	22 422 (85.9)	<.001
1	38 709 (12.2)	627 (8.0)	9413 (12.8)	16 576 (11.9)	7719 (13.7)	2941 (11.3)	
≥2	12 219 (3.9)	395 (5.0)	3525 (4.8)	4867 (3.5)	2311 (4.1)	738 (2.8)	
Tumor grade							
1	86 207 (27.2)	2207 (28.0)	25 773 (35.0)	35 830 (25.6)	12 970 (23.0)	5678 (21.8)	<.001
2	230 383 (72.8)	5669 (72.0)	47 831 (65.0)	103 875 (74.4)	43 476 (77.0)	20 423 (78.2)	
Progesterone receptor							
Negative	21 263 (7.4)	579 (8.1)	4240 (6.4)	9402 (7.5)	4372 (8.5)	1809 (7.6)	<.001
Positive	265 024 (92.6)	6543 (91.9)	61 734 (93.6)	116 691 (92.5)	46 983 (91.5)	21 945 (92.4)	

Abbreviation: NA, not applicable.

^a^ *P* values were computed using analysis of variance, Kruskal-Wallis test, or Pearson χ^2^ test.

^b^ Includes American Indian, Alaska Native, other, or unknown.

^c^ Defined as education attainment for patient residential areas and measured by matching the zip code of the patient recorded at the time of diagnosis against files derived from the 2020 American Community Survey data.

^d^ Based on the 2020 American Community Survey data, spanning years 2016 to 2020 and adjusted for 2016 inflation.

^e^ Measured by matching the state and county Federal Information Processing Standard code of the patient recorded at the time of diagnosis against 2013 files published by the US Department of Agriculture Economic Research Service.

Overall, 46.0% of patients underwent lumpectomy plus adjuvant radiotherapy, followed by 24.2% lumpectomy alone, 18.6% unilateral mastectomy, 8.6% bilateral mastectomy, and 2.6% no surgery. Younger patients were more likely to undergo bilateral mastectomy (mean [SD] age, 53.2 [10.9] years vs from 59.2 [12.4] years for unilateral mastectomy to 65.4 [12.1] years for lumpectomy). The nonsurgical management group included a higher proportion of Black patients (20.5%) compared with other treatment groups (from 10.2% with bilateral mastectomy to 15.9% with unilateral mastectomy). Similarly, patients who were uninsured (2.4%) had higher representation in the nonsurgical management group than those in other treatment modality groups (from 1.0% in the lumpectomy-only group to 1.6% in the unilateral mastectomy group), as were patients with lower incomes (median household income quartile 1, 16.0% vs 11.0%-14.8% in the other treatment groups) and low educational attainment (no high school degree quartile 1, 22.1% vs 13.8%-19.0% in the other treatment groups). In contrast, the bilateral mastectomy group included a higher proportion of White patients (79.2% vs 65.4%-74.7% in the other treatment groups) and privately insured patients (74.6% vs 42.7%-57.7% in the other treatment groups), as well as patients residing in higher-income areas (median household income quartile 4, 49.5% vs 43.2%-46.9% in the other treatment groups) and areas with higher educational attainment (no high school degree quartile 4, 31.3% vs 24.9%-27.6% in the other treatment groups) (Table).

### Trends in Nonsurgical Management and Local Treatment Modalities

**Figure 2.**
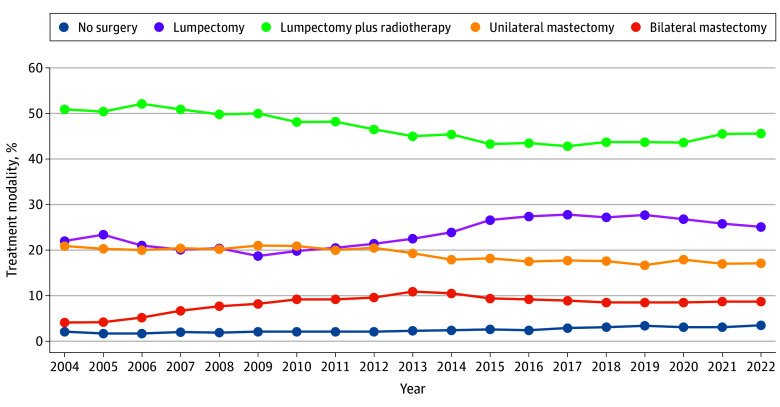
Temporal Trends in Treatment Modalities for Low-Risk, Hormone Receptor–Positive Ductal Carcinoma In Situ, 2004-2022

Figure 2 illustrates the temporal trends in no surgery and treatment strategies for low-risk, HR-positive DCIS. Although nonsurgical management remained the least frequently used treatment modality, its use increased from 2.1% in 2004 to 3.5% in 2022 (relative change per year, 4.22%; 95% CI, 3.75%-4.68%; *P* for trend < .001). Bilateral mastectomy increased from 4.1% to 8.7% (relative change per year, 1.47%; 95% CI, 1.25%-1.68%; *P* for trend < .001). Lumpectomy alone also increased from 22.0% to 25.1% (relative change per year, 1.93%; 95% CI, 1.80%-2.05%; *P* for trend < .001), whereas lumpectomy plus radiotherapy declined from 50.9% to 45.6% (relative change per year, –0.93%; 95% CI, –1.01% to –0.86%; *P* for trend < .001). Unilateral mastectomy also declined over the period from 20.9% to 17.1% (relative change per year, –1.37%; 95% CI, –1.51% to –1.23%; *P* for trend < .001).

### Trends in Endocrine Therapy Use Across Treatment Strategies

**Figure 3.**
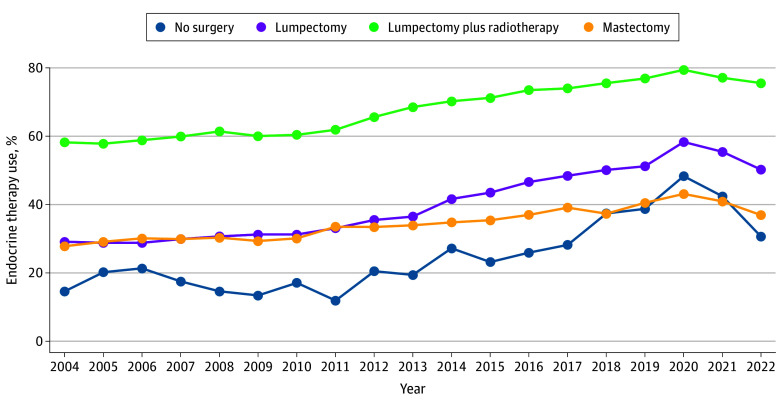
Temporal Trends in Endocrine Therapy Use Among Patients With Low-Risk, Hormone Receptor–Positive Ductal Carcinoma In Situ Across Treatment Modalities, 2004-2022

As shown in eFigure 1 in Supplement 1, overall endocrine therapy use was highest among patients treated with lumpectomy plus adjuvant radiotherapy (69.6%), followed by lumpectomy alone (43.9%), mastectomy (35.3%), and no surgery (29.2%). Patients younger than 50 years had lower endocrine therapy use than those aged 50 years or older within the no surgery (15.2% vs 31.9%) and lumpectomy alone (38.6% vs 44.6%) groups. Endocrine therapy use after lumpectomy plus adjuvant radiotherapy increased from 58.2% in 2004 to a peak of 79.4% in 2020, followed by a decline to 75.5% in 2022 (relative change per year, 1.87%; 95% CI, 1.80%-1.94%; *P* for trend < .001) (Figure 3). Similarly, endocrine therapy use after lumpectomy alone increased to 58.3% in 2020 from 29.1% in 2004 but declined to 50.2% in 2022 (relative change per year, 4.32%; 95% CI, 4.14%-4.51%; *P* for trend < .001). Endocrine therapy use after unilateral mastectomy increased from 27.8% in 2004 to a peak of 43.1% in 2020, followed by a decline to 36.9% in 2022 (relative change per year, 2.18%; 95% CI, 1.96%-2.41%; *P* for trend < .001). Among patients treated nonsurgically, endocrine therapy use increased from 14.6% in 2004 to 48.3% in 2020, then fell to 30.6% in 2022 (relative change per year, 6.78%; 95% CI, 5.94%-7.62%; *P* for trend < .001).

### Trends in Adjuvant Radiotherapy Use by Genomic Risk

Among 2626 patients from 2018 to 2022 who underwent lumpectomy and had a valid ODX DCIS score, 2188 (83.3%) scored as low risk, 276 (10.5%) as intermediate risk, and 162 (6.2%) as high risk. Adjuvant radiotherapy use was higher in the ODX high-risk stratum (73.1%) and the intermediate-risk stratum (63.9%) than in the low-risk stratum (34.5%) (eFigure 2 in Supplement 1). Among low-risk patients, radiotherapy use increased steadily over time, from 31.8% in 2018 to 40.4% in 2022 (relative change per year, 4.92%; 95% CI, 0.77%-9.25%; *P* for trend = .02). Intermediate-risk patients also showed an increase in radiotherapy use from 59.2% to 71.4% (relative change per year, 6.78%; 95% CI, 0.73%-13.20%; *P* for trend = .03). Radiotherapy use among high-risk patients remained stable over the 5-year period (relative change per year, –0.02%; 95% CI, –7.40% to 7.95%; *P* for trend > .99) (eFigure 3 in Supplement 1).

## Discussion

This cross-sectional study of a large national sample of patients with low-risk, HR-positive DCIS found that substantial shifts in local treatment strategies were observed over nearly 2 decades. While conventional treatment with lumpectomy plus adjuvant radiotherapy remained the predominant regimen, 2 additional trends have emerged: a movement toward deescalation, as reflected in increasing use of nonsurgical management, and a movement toward more aggressive or preventive strategies, as illustrated by the increase in bilateral mastectomy. These contrasting patterns highlight a growing heterogeneity in the contemporary management of low-risk DCIS and possibly better precision treatment.

Clinical adoption of nonsurgical management has been gradually increasing, reaching approximately 3.0% of low-risk patients with DCIS in the US in 2019,^11^ similar to 2.6% in our study, and further increasing to 3.5% in 2022. Importantly, this increase began before the publication of COMET and other active surveillance trial results, suggesting that factors beyond trial evidence, eg, evolving perceptions of risk, growing availability of genetic testing, and increasing interest in less intensive management among both clinicians and patients, may have contributed. Although formal guideline endorsement is limited (current National Comprehensive Cancer Network guidelines do not recommend active surveillance for DCIS), the landscape surrounding deescalation has nonetheless shifted. Emerging economic analyses have suggested that surveillance-based strategies may be cost-effective for carefully selected low-risk patients,^23^ and patient preference work (eg, LORD-PPT) has indicated that up to three-quarters of fully informed women would choose surveillance.^24^ At the same time, multiple barriers continue to constrain broader adoption of surveillance-based approaches in clinical practice and include (1) approximately 26.0% of biopsy-diagnosed DCIS that can be upstaged to invasive cancer at surgery,^25^ (2) surveillance schedules that still demand twice-yearly imaging and clinic visits,^11,15^ (3) concern about psychological burden and loss to follow-up,^26^ and (4) a label of carcinoma that can magnify anxiety and overtreatment.^27^ Collectively, these opposing pressures may help explain why no surgery, although still uncommon overall, has nonetheless emerged as a leading deescalation strategy currently under consideration for low-risk DCIS.

Our analysis revealed heterogeneity in treatment selection across sociodemographic subgroups. Nonsurgical treatment was chosen more frequently among Black patients and patients without insurance. These patterns align, in part, with findings from Poli et al,^28^ who similarly observed greater use of nonoperative management among Black and uninsured women in the southern US. In our study, however, the gradient extended beyond race and insurance status: Patients residing in areas with lower income or lower educational attainment were also more likely to undergo nonsurgical treatment. These findings suggest that economic and structural factors, beyond individual preference, may influence the selection of no surgery. In contrast, the higher rate of bilateral mastectomy among socioeconomically advantaged groups may reflect distinct psychosocial dynamics. A population-based study of women younger than 50 years with DCIS found that the rate of bilateral mastectomy increased from 11.0% in 2004 to 27.0% in 2016, with the highest uptake among younger, White, privately insured women with higher levels of income and education.^29^ These trends may be influenced by heightened anxiety about recurrence, misperceptions of contralateral breast cancer risk, and a desire for perceived treatment finality, factors that can outweigh clinical indicators in shaping treatment decisions for low-risk DCIS.^30,31^

In our cohort of patients with HR-positive DCIS, endocrine therapy use was generally low but increased in recent years, reaching up to 48.3% in 2020. Notably, patients with HR-positive DCIS treated with no surgery or lumpectomy alone had lower endocrine therapy uptake compared with those who underwent lumpectomy plus adjuvant radiotherapy. This pattern may reflect patient preferences. Interim results from the phase 3 EUROPA trial showed that radiotherapy led to better quality of life and fewer adverse events than endocrine therapy, helping to explain why many patients may prefer radiotherapy over long-term endocrine therapy.^32^ Younger patients have reported a heavier burden of adverse effects, including vasomotor symptoms, joint or muscle pain, mood changes, fertility concerns, and the inconvenience of lifelong daily medication.^33,34^ These issues have been associated with early discontinuation and nonadherence, which may explain the lower rates of endocrine therapy initiation and persistence in younger women.^35,36^ Some single-institution studies have also reported promising long-term outcomes with endocrine therapy–based active surveillance. Glencer et al^37^ found that more than half of patients with HR-positive DCIS did not develop invasive disease during a mean follow-up of 7.4 years under magnetic resonance imaging monitoring with endocrine therapy. Their cohort, though small (n = 71), had a mean age of 53.8 years and included nearly 50.0% premenopausal women, highlighting the applicability of these findings to younger populations. However, this protocol, magnetic resonance imaging–based surveillance plus endocrine therapy, differs substantially from the nonsurgery group derived from NCDB, which lacked standardized imaging or structured follow-up. Collectively, these observations highlight the importance of individualized counseling to address patient-specific concerns and to support informed decision-making, particularly for younger women who may be candidates for surveillance-based strategies. Although endocrine therapy use steadily increased through 2020, a subsequent decline was observed across treatment modalities. This trend may reflect several converging factors: (1) During the COVID-19 pandemic, endocrine therapy may have been used as a temporary bridge in a subset of patients experiencing surgical delays, potentially contributing to the transient rise in endocrine therapy use observed in 2020; (2) once surgical services returned to normal, this interim strategy receded; and (3) shifts in patient preferences, fewer in-person visits, and nonadherence further contributed to the reduction in endocrine therapy use.^38,39^

We show that adjuvant radiotherapy was guided by ODX DCIS scores, ranging from 34.5% in the low-risk group to 73.1% in the high-risk group. This finding aligns with prospective data showing that results from genomic testing meaningfully influence radiotherapy decisions, modifying recommendations in nearly one-third of patients with DCIS and helping to estimate local recurrence risk.^40,41^ While not universally applied, radiotherapy seems to be increasingly tailored to molecular risk. We observed a persistent, and even increasing, use of radiotherapy among some low-risk patients. This pattern suggests that factors beyond genomic risk stratification continue to influence clinical decision-making. In particular, the increase in radiotherapy use among patients with low ODX scores from 2018 to 2022 may reflect the lag between the availability of genomic assays and their full integration into routine practice, as well as variability in clinician familiarity with these tools and the absence of standardized score thresholds for safely omitting radiotherapy. These broader influences, including variation in clinician comfort with genomic assays, institutional practice patterns, and patient preference for perceived treatment finality, may collectively contribute to radiotherapy use, even in patients classified as low risk by ODX DCIS testing.^41^ A concerted effort to better align radiotherapy recommendations with validated risk profiles may help minimize unnecessary interventions. As the field advances toward risk-adapted, patient-centered care, these trends suggest both promising developments and missed opportunities. While enthusiasm for deescalation is growing, implementation may have outpaced the availability of supportive tools (eg, reliable biomarkers, clear clinical definitions of low-risk disease, robust surveillance systems). Addressing these gaps could be critical to advancing individualized management strategies for low-risk DCIS.

### Limitations

This study was subject to the inherent limitations of a retrospective, registry-based study design. First, nonoperative cases were identified using NCDB surgery and radiation variables, following criteria adapted from previous NCDB and Surveillance, Epidemiology, and End Results studies. Because these variables cannot distinguish protocol-driven active surveillance from unstructured watchful waiting, treatment refusal, delayed surgery, limited life expectancy, or socioeconomic barriers, the nonoperative group in this study (labeled as no surgery or nonsurgical management) represented a heterogeneous set of clinical scenarios and did not reflect true surveillance-based care. Although excluding patients with less than 12 months of follow-up reduced the likelihood of misclassifying delayed surgery as nonsurgical management, misclassification remains possible, particularly because frailty and competing mortality risks were not fully captured. The NCDB lacks information on imaging frequency, surveillance intensity, patient preference, and clinician rationale. As such, the data cannot differentiate structured surveillance protocols from unstructured watchful waiting. High-grade DCIS was intentionally excluded to align with contemporary surveillance trials, improving comparability but limiting generalizability to low-risk, HR-positive disease.

Second, endocrine therapy is susceptible to underrecording in the NCDB because oral medications are not always captured. This underascertainment may cause endocrine therapy use to appear lower than it truly is, especially in the nonoperative group. In contrast, surgical procedures, when documented, are unlikely to be miscoded, although a small chance exists for undocumented procedures.

Third, because the NCDB contains very large sample sizes, even small differences may be statistically significant despite representing minimal clinical differences. Therefore, statistically significant findings in this study should be interpreted with appropriate caution.

Fourth, molecular risk stratification was feasible in approximately 1.0% of the patients with an ODX DCIS score in recent years, limiting the representativeness of genomic analyses. Radiotherapy patterns among patients with low ODX scores should therefore be interpreted cautiously, as incomplete adoption of genomic tools and variation in score interpretation may influence treatment decisions.

Finally, DCIS treatment patterns after 2020 may have been influenced by COVID-19–related service disruptions and interim practice changes. Because overall survival was not a prespecified study end point and cannot be reliably interpreted in the context of strong confounding for nonoperative management, survival comparisons should be viewed as exploratory. These limitations should be considered when interpreting the actual clinical management patterns described in this study.

## Conclusions

This cross-sectional study of low-risk, HR-positive DCIS in the US found that the use of nonsurgical management increased over time as a deescalation approach. This strategy was more frequently selected by Black patients and patients without insurance. In contrast, bilateral mastectomy more than doubled during the study period, with the highest rates observed among younger, White, and privately insured patients and those living in higher-income and higher-education areas. These divergent trends suggest that treatment selection may be influenced in part by sociodemographic context. The growing heterogeneity in clinical DCIS management highlights the need for precision prevention, including refined risk stratification, equitable access to genomic and imaging tools, and shared decision-making frameworks, that aligns the type and intensity of intervention with each patient’s risk profile and personal preferences to advance individualized management of low-risk DCIS.

## Notes

Supplement 1. **eFigure 1. **Endocrine Therapy Use Across Treatment Modalities and Age Groups Among Patients With Low-Risk, Hormone Receptor–Positive Ductal Carcinoma In Situ **eFigure 2. **Radiotherapy Use in Low-Risk, Hormone Receptor–Positive Ductal Carcinoma In Situ, by Oncotype DCIS Score Category **eFigure 3. **Trends in Radiotherapy Use in Low-Risk, Hormone Receptor–Positive Ductal Carcinoma In Situ From 2018 to 2022, by Oncotype DCIS Score Category

Supplement 2.
Data Sharing Statement
