# Supplementary material for: Trends in Nonsurgical Management for Low-Risk, Hormone Receptor–Positive Ductal Carcinoma In Situ
Source: JAMA Netw Open. 2026 Feb 10;9(2):e2558248. doi: 10.1001/jamanetworkopen.2025.58248 (PMC12892143; doi:10.1001/jamanetworkopen.2025.58248)
Supplement: Supplement 1. — eFigure 1. Endocrine Therapy Use Across Treatment Modalities and Age Groups Among Patients With Low-Risk, Hormone Receptor–Positive Ductal Carcinoma In Situ eFigure 2. Radiotherapy Use in Low-Risk, Hormone Receptor–Positive Ductal Carcinoma In Situ, by Oncotype DCIS Score Category eFigure 3. Trends in Radiotherapy Use in Low-Risk, Hormone Receptor–Positive Ductal Carcinoma In Situ From 2018 to 2022, by Oncotype DCIS Score Category [file jamanetwopen-e2558248-s001.pdf]

Supplemental Online Content

Matsui Y, Freeman JQ, Poland S, et al. Trends in nonsurgical management for low-risk, hormone receptor–positive ductal carcinoma in situ. *JAMA Netw Open*. 2026;9(2):e2558248. doi:10.1001/jamanetworkopen.2025.58248

eFigure 1. Endocrine Therapy Use Across Treatment Modalities and Age Groups Among Patients With Low-Risk, Hormone Receptor–Positive Ductal Carcinoma In Situ
eFigure 2. Radiotherapy Use in Low-Risk, Hormone Receptor–Positive Ductal Carcinoma In Situ, by Oncotype DCIS Score Category
eFigure 3. Trends in Radiotherapy Use in Low-Risk, Hormone Receptor–Positive Ductal Carcinoma In Situ From 2018 to 2022, by Oncotype DCIS Score Category

This supplemental material has been provided by the authors to give readers additional information about their work.

© 2026 Matsui Y et al. JAMA Network Open.

eFigure 1. Endocrine Therapy Use Across Treatment Modalities and Age Groups Among Patients With Low-Risk, Hormone Receptor–Positive Ductal Carcinoma In Situ

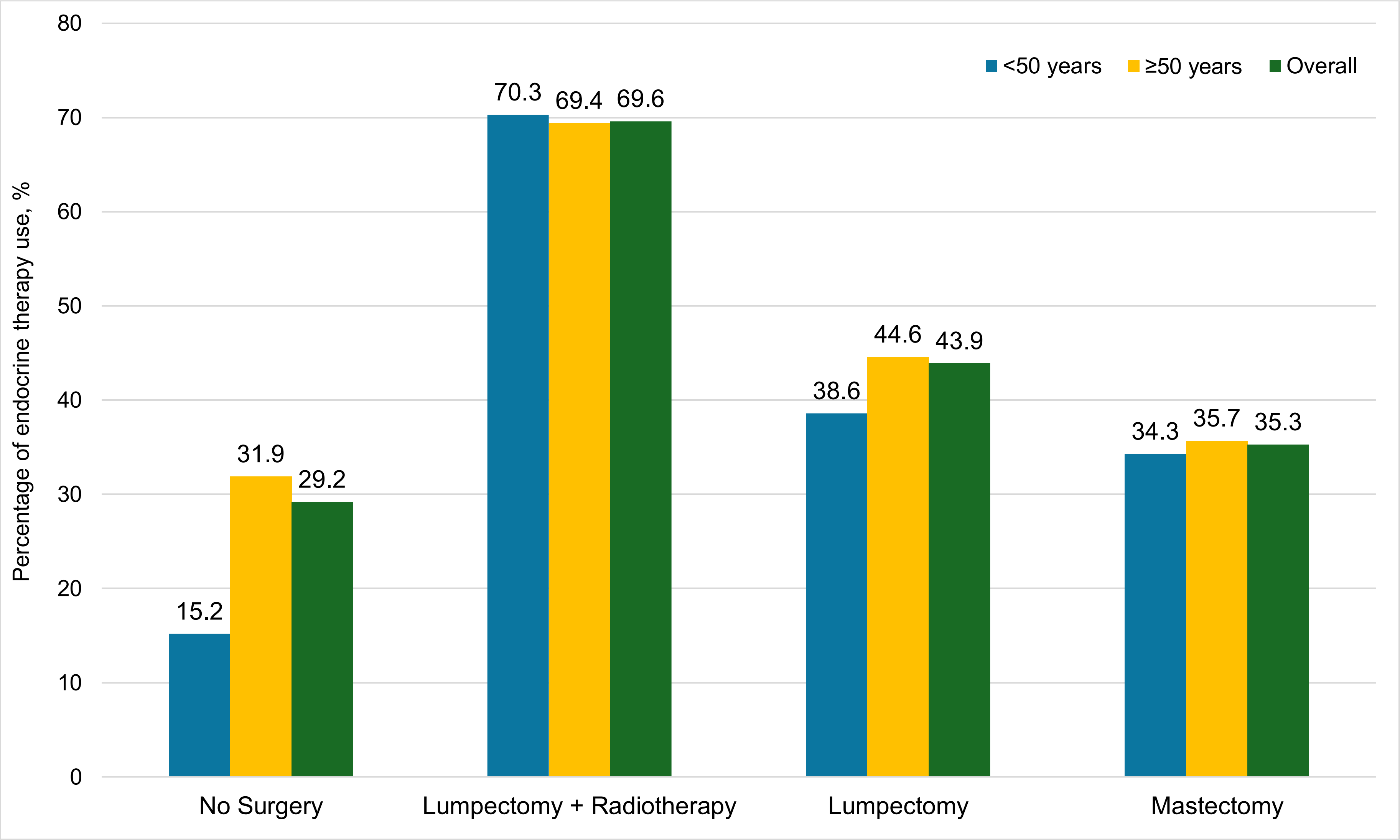

© 2026 Matsui Y et al. JAMA Network Open.

eFigure 2. Radiotherapy Use in Low-Risk, Hormone Receptor–Positive Ductal Carcinoma In Situ, by Oncotype DCIS Score

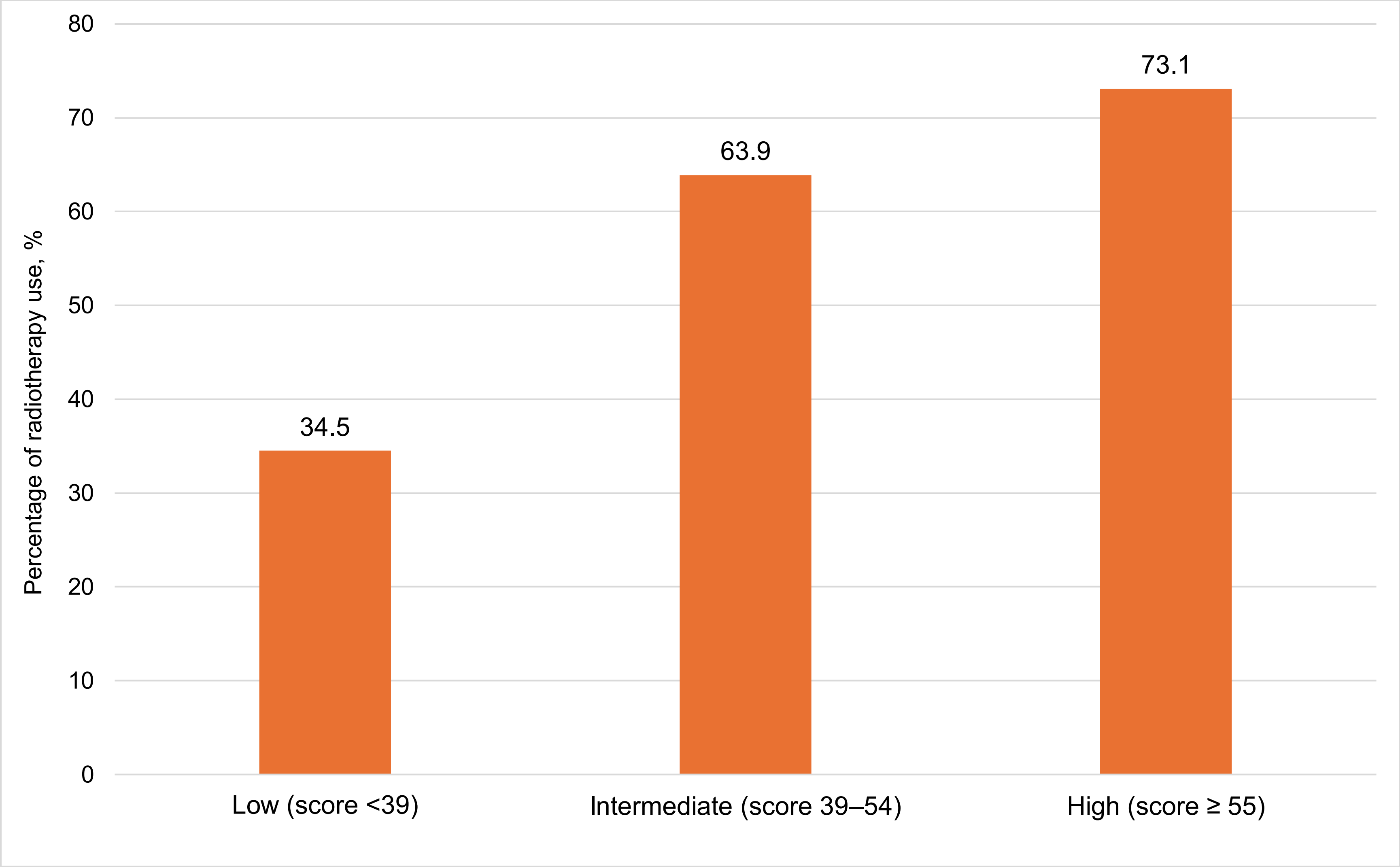

© 2026 Matsui Y et al. JAMA Network Open.

eFigure 3. Trends in Radiotherapy Use in Low-Risk, Hormone Receptor–Positive Ductal Carcinoma In Situ From 2018 to 2022, by Oncotype DCIS Score Category

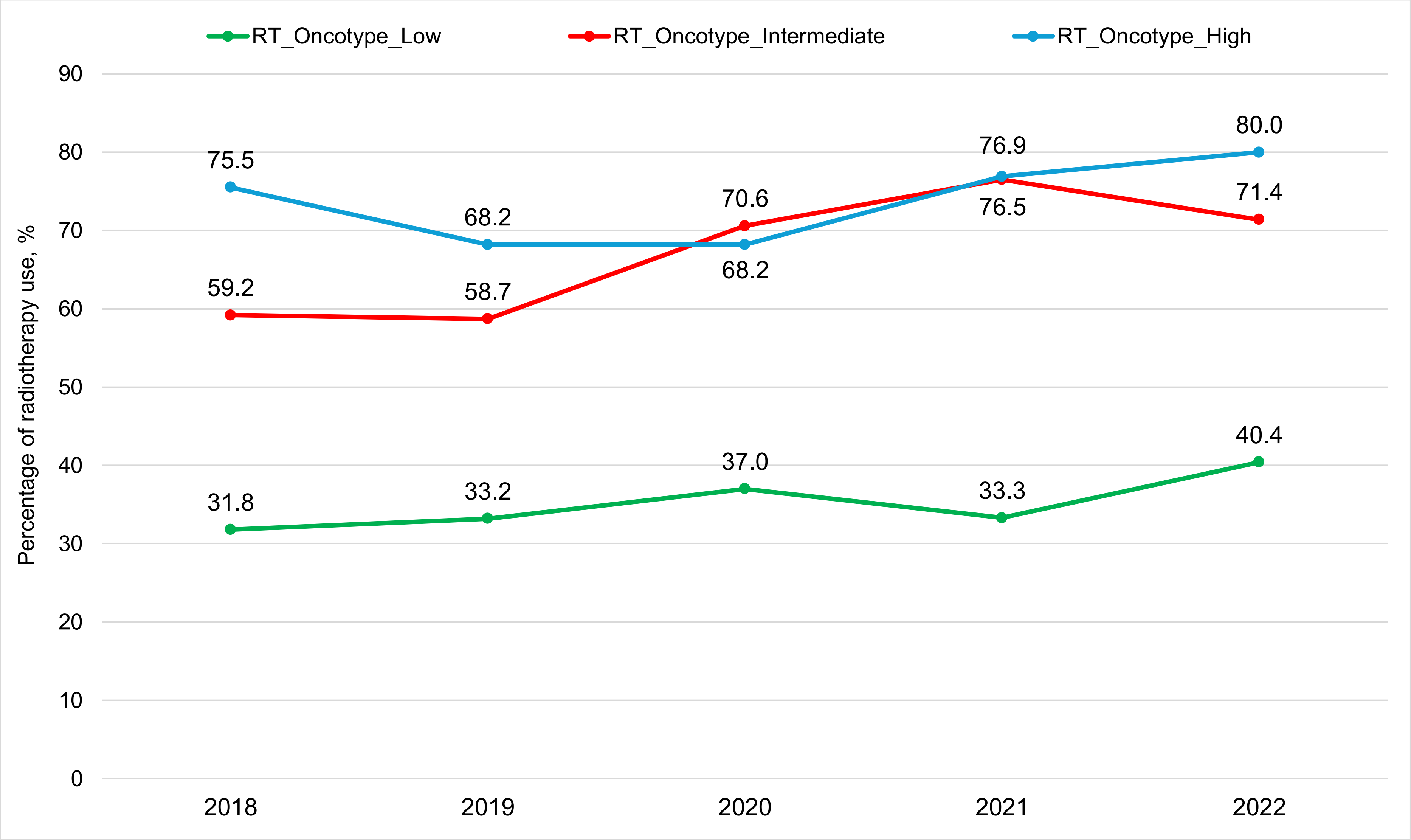

© 2026 Matsui Y et al. JAMA Network Open.
