# Supplementary material for: Trends in Nonsurgical Management for Low-Risk, Hormone Receptor–Positive Ductal Carcinoma In Situ
Source: JAMA Netw Open. 2026 Feb 10;9(2):e2558248. doi: 10.1001/jamanetworkopen.2025.58248 (PMC12892143; doi:10.1001/jamanetworkopen.2025.58248)
Supplement: Supplement 2. — Data Sharing Statement [file jamanetwopen-e2558248-s002.pdf]

Data Sharing Statement

Data
Data available: No

Additional Information
Explanation for why data not available: Data Data available: Yes Data types: De-identified patient data How to access data: This study used data collected from the 2004-2022 National Cancer Database. Investigators associated with Commission on Cancer-accredited cancer programs can request the data by submitting a Participant User Data File application directly to the American College of Surgeons through https://www.facs.org/quality-programs/cancer-programs/national-cancer-database Supporting Documents Document types: None Additional Information Who can access the data: Anyone requesting the data Types of analyses: Any purpose Mechanisms of data availability: Requires a Participant User Data File application to the American College of Surgeons and a signed data use agreement according to the National Cancer Database https://www.facs.org/quality-programs/cancer-programs/national-cancer-database/puf/
